# Morphophysiological and transcriptomic analyses during the development of microspores and megaspores in *Orobanche coerulescens*

**DOI:** 10.3389/fpls.2025.1540594

**Published:** 2025-03-26

**Authors:** Kelin Cui, Jingyi Liu, Yuanyuan Xie, Yaqin Xiao, Yuxin Tian, Lijuan Jiang, Yandong Niu

**Affiliations:** ^1^ College of Life and Environmental Sciences, Central South University of Forestry and Technology, Changsha, Hunan, China; ^2^ Hunan Academy of Forestry, Changsha, Hunan, China; ^3^ Hunan Dongting Lake Wetland Ecosystem Positioning Observation and Research Station, Changsha, Hunan, China; ^4^ Field Observation and Research Station of Dongting Lake Natural Resource Ecosystem, Ministry of Natural Resources, Changsha, Hunan, China; ^5^ International Technological Cooperation Base for Ecosystem Management and Sustainable Utilization of Water Resources in Dongting Lake Basin, Changsha, Hunan, China

**Keywords:** megaspores, microspores, reproductive biology, histological analysis, *Orobanche coerulescens*, phytohormones, transcriptomics, metabolic pathways

## Abstract

**Introduction:**

*Orobanche coerulescens* is a parasitic plant considered as a malignant weed due to its harmful effects on crops. However, its richness in high-value secondary metabolites makes it a significant medicinal resource. The development of microspores and megaspores is essential for sexual reproduction in plants but research on this aspect of *O. coerulescens* is lacking.

**Methods:**

This study aimed to systematically observe the developmental processes of microspores and megaspores in *O. coerulescens* using microscopic techniques. We measured the levels of soluble sugar, starch, and phytohormones during different developmental stages. We also investigated the key regulatory genes in the metabolic pathways of phytohormones that are closely related to the development of microspores and megaspores using transcriptome sequencing technology.

**Results and discussion:**

The findings revealed that the flower development process of *O. coerulescens* could be categorized into six stages. Mature pollen was tricellular, with downy ornamentation and pores on the outer wall. The embryo sac was the monosporangiate polygonum type, and the ovule was inverted. The megaspores developed and matured about 15 days later than the microspores. The soluble sugar level of the flower buds decreased initially and then increased during development, whereas the starch level showed an opposite trend. The levels of strigolactone, auxin, and gibberellins gradually increased throughout the development process. The key genes regulating phytohormone synthesis during the development of microspores and megaspores were identified as *ALDHs* (Aldehyde Dehydrogenases). In contrast, the key genes regulating phytohormone signaling included *TIR1* (Transport Inhibitor Response 1) and *IAA3* (Indole-3-acetic Acid Inducible 3), and the key TF was *ARF5* (AUXIN RESPONSE FACTOR 5). The findings of this study enhanced the understanding of *O. coerulescens* biology, providing theoretical references for regulating its reproduction, implementing biological control measures, maintaining its population, and optimizing resource utilization.

## 1 Introduction

*Orobanche coerulescens* is a biennial or perennial phytoparasite of the genus *Orobanche* in the family Orobanchaceae. It parasitizes *Artemisia selengensis*, featuring multiple flowers arranged in spikes and bisexual flowers (Wu et al., 1998). *Orobanche* is one of the largest genus of parasitic plants, comprising about 200 species with a wide global distribution.

*O. coerulescens* relies entirely on the host to obtain nutrients and water, severely damaging the host plant by reducing the aboveground biomass and leaf chlorophyll content (Parker, 2009; Westwood et al., 2010). Each *O. coerulescens* plant can produce hundreds of thousands of seeds with a diameter of less than 1 mm per plant (Eizenberg et al., 2012), which remain dormant in the soil for up to 15 years without being induced by external stimuli (Francisca and Luis, 1999). Its unique parasitic characteristics make it difficult to eradicate in the field, affecting a wide range of crops. Global crop losses due to *Orobanche* infestation are estimated at $1.3–$2.6 billion annually (Das et al., 2020). Controlling *O. coerulescens* in agricultural systems remains a huge challenge. At present, selecting resistant varieties and exploring the biochemical pathways in crops that confer resistance to *Orobanche* is an effective method (Abbes et al., 2020; En-nahli et al., 2023). Using exogenous stimuli or non-host plants and their extracts to trap and kill *Orobanche* seeds, also known as suicidal germination, is become a new way to control it (Zwanenburg et al., 2016). The exchange of small RNAs (sRNAs) between crops and parasitic plants has been discovered in recent years, and the method of protecting crops by delivering sRNAs to parasitic plants called host-induced gene silencing, is also a promising method for controlling *Orobanche* in the future (Zainali et al., 2024). However, the effectiveness of these strategies may vary, and ongoing research is needed to develop more targeted and sustainable management approaches.

However, parasitic plants are not just harmful plants; they play crucial roles in ecosystems as well. Parasitic plants often exert top–down control on plant communities. By parasitizing dominant species, they reduce their competitive advantage, thus facilitating the coexistence of less competitive species (Press and Phoenix, 2005). Parasitic plants can affect nutrient cycling by altering the flow of carbon, nitrogen, and other essential nutrients in ecosystems. They mobilize nutrients that otherwise remain locked in their hosts, indirectly affecting carbon sequestration in terrestrial ecosystems and redistributing them into the soil and benefiting other plants in the community (Quested et al., 2005; Grewell, 2008; Demey et al., 2014). They reduce the energy capture potential of their host plants and redirect this energy into their own growth and reproduction, influencing the food web and indirectly affecting carbon sequestration in terrestrial ecosystems (March and Watson, 2007; Watson, 2009; Mellado et al., 2016). In some ecosystems, parasitic plants help facilitate ecological succession in disturbed or early-successional habitats by disturbing established plant communities, thereby creating space for other species to establish (Griebel et al., 2017; Skay et al., 2021; Gougherty et al., 2024). Studies have shown that *Orobanche* genus plants obtain nutrients from their host plants through haustoria. This parasitic process significantly reduces the photosynthetic efficiency, biomass, and seed yield of the host plants, thereby altering the structure of surface plant communities. In response, host plants may resist parasitism by modifying their root exudates or activating defense mechanisms (Joel et al., 2007). Meanwhile, plants of the *Orobanche* genus are rich in various natural products. In recent years, an increasing number of natural products have been discovered in *O. coerulescens*, which have analgesic effects, antimicrobial activities, blood pressure–lowering and platelet aggregation–improving effects, memory-enhancing effects, and other functions (El-Shabrawy et al., 1989; Isacchi et al., 2011; Gao et al., 2015; Scharenberg and Zidorn, 2018). *O. coerulescens* is a traditional Chinese medicine widely used to treat back and knee pain and has a long history in northern China (Zhang et al., 2020). Therefore, resource utilization of *O. coerulescens* is a worthwhile research direction in the future.

Sexual reproduction is crucial for maintaining population and genetic diversity (Wilson and Zhang, 2009). Reproductive strategies of parasitic plants are influenced by their habitats and host plants. For example, *Cuscuta australis* lacks the gene for the functional FLOWERING LOCUS T (FT) regulator of flowering and appears to eavesdrops on the FT signal of the host plant in order to synchronize its flowering with the flowering of the host, thus favoring its reproduction (Shen et al., 2020). Reproductive strategies of parasitic plants are also influenced by host species, compared to *Phoradendron californicum* parasitized on *Prosopis velutina*, *P. californicum* parasitized on *Senegalia greggii* is larger in size with larger, more densely packed flowers that produced more nectar and pollen (Yule and Bronstein, 2018). Other studies in this project have shown that the flowering and fruiting periods of *O. coerulescens* in the wetlands of southern China occur approximately three months earlier compared to those in the semi-arid regions of northern China to avoid the flood season. Like other holoparasitic plants(such as *C. japonica*), *O. coerulescens* steals all the water and nutrients from its host through a key organ called haustorium, which exhibits a high degree of parasitic specialization (Parker, 2009; Yoshida et al., 2016). At the same time, the germination of *O. coerulescens* seeds is also highly specialized, triggered by specific chemical signals (such as strigolactone, SL) released from host plants, a characteristic that is commonly observed in many parasitic plants, including Striga, which ensures that the seeds are only activated when a suitable host is available (Yoshida et al., 2016). The reproductive strategy of the *Orobanche* genus exhibits remarkable adaptability. Not only can it utilize both self-pollination and cross-pollination to enhance reproductive efficiency, but the flowers of *Orobanche* plants are also often brightly colored and produce volatile compounds to attract pollinators. Additionally, they produce a large number of tiny seeds capable of lying dormant in the soil for many years, awaiting the presence of a host. Such a combination of traits is rare among parasitic plants and other plant species (Tóth et al., 2016; Nishimura and Takayama, 2023). The development of microspores and megaspores is one of the most important part of sexual reproduction, which is critically governed by intricate genetic and hormonal regulatory networks (Barro-Trastoy et al., 2020). Auxin, the most well-studied phytohormone, is involved in almost all reproductive processes (Zhang et al., 2023). During pistil development, the peak concentration of indole-3-acetic acid (IAA) specifies the initiation site of ovule primordia (Nole-Wilson et al., 2010). SL is a phytohormone that functions as both an exogenously secreted signaling molecule and an endogenous phytohormone (Guercio et al., 2023). In recent years, the functions of SL and its interactions with other phytohormone signaling pathways have been increasingly explored (Omoarelojie et al., 2019). Carbohydrates serve as essential energy sources and structural components in developing plant microspores and megaspores. In *Zea mays* (maize), starch biosynthesis during pollen maturation is associated with altered gene expression patterns; reduced sugar and starch synthesis can lead to pollen abortion (Datta et al., 2002). Environmental stresses can disrupt these carbohydrate dynamics, leading to male sterility due to reduced sugar and starch levels in anthers (De Storme and Geelen, 2014). Overall, the precise regulation of sugar and starch metabolism is fundamental to the successful development of both microspores and megaspores in plants.

Using transcriptomics helps us understand plant reproductive development. Researchers have conducted a combined analysis of transcriptome and nontargeted metabolomes to reveal the mechanism of *Cymbidium sinense* ovule development, providing a valuable basis for further understanding the processes governing *C. sinense* ovule development and formation (Zeng et al., 2022). Multiple key canonical floral integrators and pathways have been identified through transcriptome differential expression analysis. This provided insights into the molecular regulatory mechanisms of reproductive development of *Tripidium ravennae*, including genome annotation, functional genomic features, gene family progression, comparative genomics, and precision breeding (Maren et al., 2021). Identifying candidate genes through transcriptome profiling also helps understand the molecular regulatory mechanisms of genes related to the development of microspores and megaspores (Ji et al., 2022).

Currently, a few studies have investigated the reproductive development of *O. coerulescens* (Prider, 2015), which includes individual morphological development (El-Taher and Mohamed, 2022), the growth and anatomical structure of their haustorium, the process of invading host plants (Yoshida et al., 2016; Al-Joboury and Aliwy, 2021), the anatomical structure of flowers, the microstructure of pistils and stamens (Konarska and Chmielewski, 2020; Ruraż and Piwowarczyk, 2022), and the morphology and classification of pollen and seeds (Piwowarczyk et al., 2014, 2015; Pavlova and Bani, 2019). However, comprehensive research on the developmental processes and physiological and biochemical characteristics of microspores and megaspores of *O. coerulescens* is currently lacking. This study systematically examined the developmental phenology and external morphology of inflorescences and florets, as well as the development of microspores and megaspores. The study measured and analyzed the levels of soluble sugar, starch, and major phytohormones during development. This study used transcriptome sequencing technology to identify differentially expressed genes during microspore and megaspore development, screening key regulatory genes involved in phytohormone synthesis and signal transduction closely related to megaspore and microspore development. These findings enriched the foundational data for *O. coerulescens* research, laying the groundwork for deeper exploration of its reproductive strategies. This study also provided theoretical guidance for further research and the development of effective resource utilization measures.

## 2 Materials and methods

### 2.1 Plant material collection

Wild *O. coerulescens* samples were obtained from the Hengling Lake Provincial Nature Reserve, Yueyang City, Hunan Province, a region with a subtropical monsoon climate (average temperature: 17℃, annual average precipitation: 1429.3 mm, and annual sunshine: 1400–2000 h). The area is classified as a wetland, with shrublands and grasslands growing on sandy soil. The sampling points were submerged by lake water during the flood season from late June to October. The host plant *A. selengensis* was collected along with *O. coerulescens* samples and cultivated in acrylic flowerpots filled with approximately 15 cm of sandy soil. Seeds of the wild broomrape collected were grafted onto the roots of *A. selengensis*, and the potted plants were installed within the experimental area of Central South University of Forestry and Technology as supplementary samples for the field samples.

### 2.2 Morphological observation of inflorescence and floret development

Field surveys and pot observations were conducted over two consecutive years, spanning from spring 2022 to summer 2023. This was done to determine the appropriate time for field sample collection, considering the developmental status of indoor potted plants. Once the seeds broke through the soil, field observation and sampling commenced, documenting their developmental stages at 7-day intervals until the end of the *O. coerulescens* flowering stage. The collected samples were examined under a stereomicroscope (Motic, SM7, CHN), and photographed. Additionally, measurements of the external morphological features of the flower organs were recorded using an electronic vernier caliper (Deli, DL91150B, CHN).

### 2.3 Anatomical observation of microspores and megaspores

Fresh samples of *O. coerulescens* inflorescences were collected at six developmental stages. Promptly immersed in a 50% FAA (Formalin-Aceto-Alcohol) fixation solution and fixed for at least 24 h before storing for subsequent processing. Trim the sample to 1cm, using standard paraffin sectioning techniques to prepare slices of 8 μm thickness (Chen et al., 2016), employing 0.5% hematoxylin and 1% eosin staining (Feldman and Wolfe, 2014). Five samples from each stage and five sections per sample were selected. The cell and tissue structural features during the development of microspores and megaspores were examined and photographed under the optical microscope (Leica DM500; Germany). *O. coerulescens* pollens were collected during the flowering stage and observed and photographed using SEM (JEOL, JSM-7900F; JPN). For each sample, 10 representative fields of view were selected, magnified approximately 500 times to observe the overall appearance of pollen grains, magnified approximately 2500 times to observe pollen morphology features such as the germination groove, and magnified approximately 10,000 times to observe the surface texture of the pollen. The six developmental stages were defined according to the morphological changes of inflorescences and florets: F1, budding stage; F2, bud enlargement stage; F3, bud elongation stage; F4, corolla discoloration stage; F5, corolla half-open stage; and F6, corolla complete-open stage.

### 2.4 Determination of phytohormones and carbohydrate levels

To determine the soluble sugar and starch levels, 0.5 g of floret samples stored at -80℃ from six developmental stages were analyzed using the anthrone method (Wu et al., 2020). The levels of five endogenous plant hormones, namely IAA, GA, ZT, ABA, and SL, were determined using high-performance liquid chromatography (Shimadzu, LC-20A, JPN). Each group included three biological replicates (Qi et al., 2021; Chen et al., 2022).

### 2.5 RNA extraction, cDNA library construction, and transcriptome sequencing

Floret samples from the 6 developmental stages were sampled (18 samples, with 3 biological replicates each stage) were preserved at -80°C for RNA extraction using the Quick RNA extraction kit (Tiangen Biochemical Technology Co. Ltd., Beijing, China). The RNA concentration and quality were determined using NanoDrop 2000 (Thermofisher, USA). Then, the mRNA was enriched after removing rRNA using the Ribo-Zero Magnetic Kit (EPICENTRE, MRZPL116, US). The cDNA library was built based on enriched mRNA with NEBNext Ultra II RNA Library Prep Kit for Illumina (NEB, E7760L, USA) following the manufacturer’s protocols. The cDNA was stored at −80°C for sequencing and reverse transcriptase–polymerase chain reaction (RT-PCR) experiment. The RNA-seq library was sequenced on an Illumina HiSeq4000 instrument by Gene Denovo Biotechnology Co., Guangzhou, China. In order to get high quality clean reads, original reads were filtered by fastp (version 0.18.0). The parameters were as follows: removing reads containing adapters; removing reads containing more than 10% of unknown nucleotides (N); removing low quality reads containing more than 50% of low quality (Qvalue ≤ 20) bases.

### 2.6 *De Novo* assembly, Unigenes annotation, and DEG analysis

The transcriptome datasets were deposited in the National Center for Biotechnology Information database under a BioProject ID. Transcriptome *de novo* assembly was performed with clean reads filtered from the raw reads by removing adapters, unknown nucleotides (>10%), and low-quality reads (Q values ≤ 10). Then, FastQC (http://www.bioinformatics.babraham.ac.uk/projects/fastqc/) was used to verify sequence quality, including the Q20, Q30, and GC contents of clean reads. Due to the unavailability of the *O. coerulescens* genome information, clean reads from 18 samples were combined for *de novo* transcriptome assembly using the reference-genome-independent Trinity method (Grabherr et al., 2011). First, short, clean reads of a certain length were combined with overlap to form longer contigs by inchworm. Second, based on their paired-end information, clean reads were mapped back to the corresponding contigs using Chrysalis. Finally, the paths taken by reads and pairs of reads were analyzed using Butterfly. The finished sequences of the transcripts were defined as Unigenes. The Blastx program was used to annotate all assembled Unigenes (http://www.ncbi.nlm.nih.gov/BLAST/) with a threshold E value <0.00001 to the Nr (http://www.ncbi.nlm.nih.gov), Swiss-Prot protein (http://www.expasy.ch/sprot/), KEGG (http://www.genome.jp/kegg), KOG, and GO databases (http://www.geneontology.org). The expression of unigenes was normalized to RPKM (Reads Per Kilobase per Million mapped reads) values, and DEGs were identified among samples or groups using the edgeR software with |fold change| ≥2 and FDR (False Discovery Rate) <0.05 (http://www.r-project.org/). Next, GO and KEGG enrichment analyses were carried out for all DEGs, and hypergeometric tests with P ≤ 0.05 as a threshold were used to determine the significant enrichment of GO terms and KEGG pathways.

### 2.7 Co-expression network analysis of transcriptome

To determine the interaction relationship of the candidate proteins, we used the STRING protein interaction database (http://string-db.org) to construct protein–protein interaction (PPI) network with default parameters. The candidate protein set from this study was then mapped to homologous proteins in *Arabidopsis thaliana*. Based on the interaction relationship, a preliminary co-expression network was obtained. Afterward, the MCODE (Molecular Complex Detection) app (https://apps.cytoscape.org/apps/mcode) of Cytoscape software (https://cytoscape.org/) was used to obtain the most densely connected subnetworks. Finally, we obtained the final regulatory network.

### 2.8 Quantitative RT-PCR validation

Eight DEGs were validated by quantitative RT-PCR (qRT-PCR). RNA was extracted, denatured, and reverse-transcribed into first-strand cDNA using a HiScript II 1st Strand cDNA Synthesis Kit (Tiangen Biochemical Technology Co. Ltd.). PCR was performed using a Step One Plus Real-Time PCR System (Thermofisher, StepOnePlus, USN). The relative gene expression levels were normalized using the 2^-ΔΔCT^ method, with tubulin (TUA6) and actin-11 (ACT11) as the internal reference genes. Each sample included three biological replicates.

## 3 Results

### 3.1 Morphological changes in inflorescences and florets of *O. coerulescens*

**Figure 1:**
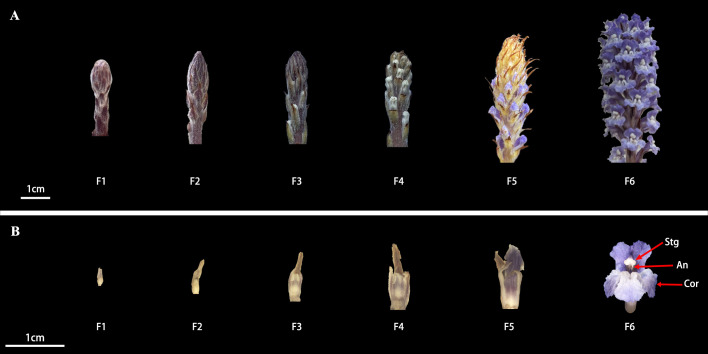
Morphological changes in inflorescences and florets of *O. coerulescens* at different developmental stages. **(A)** External morphology of inflorescences at different stages. **(B)** External morphology of florets at different stages. An, Anther; Cor, corolla; Stg, stigma.

*O. coerulescens* typically emerge from the soil in mid-February, featuring purple inflorescences with completed flower bud differentiation and bolts in late February. This study monitored the development of inflorescences and florets from the moment they emerged from the soil following flower bud differentiation to the time they reached full bloom. *O. coerulescens* features an endless spike-like inflorescence, with florets developing progressively from the base to the tip. Based on the changes in inflorescence (**Figure 1A**) and floret (**Figure 1B**) morphology, *O. coerulescens* flower development is categorized in the following six stages.

Budding stage (F1): The floret length is <3 mm, the inflorescence is predominantly purple, the plant is covered with white glandular hairs, the inflorescence tip is blunt, and the light-yellow buds are completely hidden by bracts.
Bud enlargement stage (F2): The floret length is between 3 and 7 mm, the inflorescence tip turns from blunt to pointed, the buds start to enlarge and extend, yet they remain wrapped in purple bracts, and the small flowers are completely unseen.
Bud elongation stage (F3): The floret length is between 7 and 15 mm, the purple bracts gradually turn yellow, the buds continue to expand and lengthen, the bracts become less compact, and faint yellow buds become visible.
Corolla discoloration stage (F4): The floret length is between 15 and 19 mm, the buds extend further and break through the bracts, revealing the corolla, the top of the corolla changes from yellow to blue, and the bud becomes looser due to swelling at its base.
Corolla half-open stage (F5): The floret length is between 19 and 21 mm, the top of the corolla turns deep blue-purple, and the middle section gradually bends and opens slightly, showing a hint of stamen and anecdotally opening flowers.
Corolla complete-open stage (F6): The floret length is >21 mm, and the corolla opens fully and enters its peak flowering phase, with numerous flowers in full bloom.

### 3.2 Development of microspores and megaspores of *O. coerulescens*

Florets at various developmental stages were collected for paraffin sectioning, and mature pollen was collected for scanning electron microscopy (SEM) observation to investigate the microstructural development of microspores and megaspores of *O. coerulescens*.

**Figure 2:**
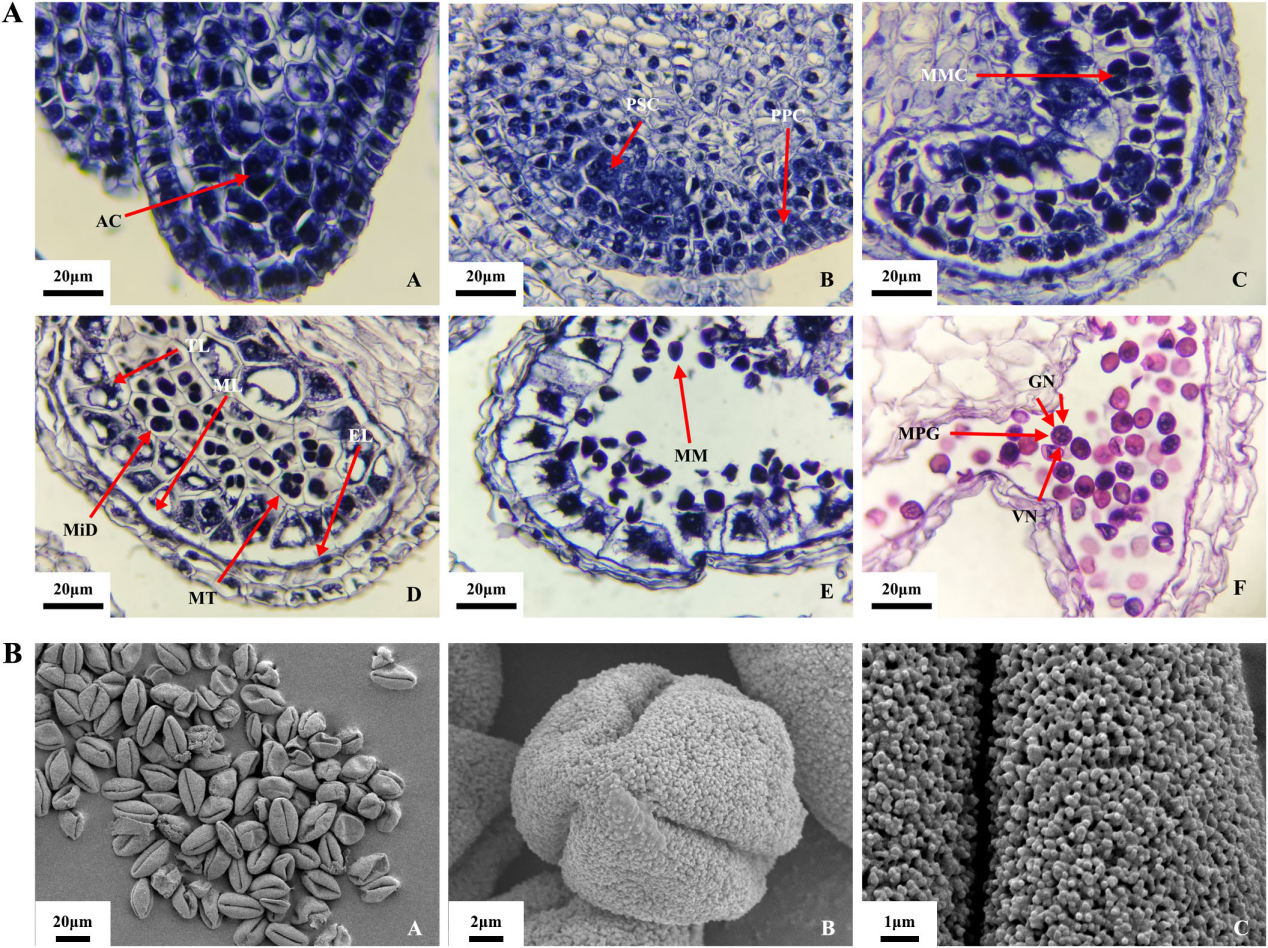
Microspore development process in *O. coerulescens*. **(A)** Microstructure of microspore at different development stages. **(B)** Surface submicroscopic structure of mature pollen. AC, Archesporial cell; PSC, primary sporogenous cell; PPC, primary parietal cell; MMC, microspore mother cell; MiD, microspore dyad; MT, microspore tetrad; EL, endothecium layer; ML, middle layer; TL, tapetum layer; MM, mononuclear microspore; MPG, mature pollen grain; VN, vegetative nucleus GN, generative nucleus.

Microscopic observation showed four anthers in the *O. coerulescens* floret, each containing four microsporangia. The microspore developed through the following stages (**Figure 2A**).

Archesporial cell stage (**Figures 2A-A**): At the beginning of another development, the structure was relatively simple. The outer epidermal cells were located below the meristematic tissue. At this time, the anther was ellipsoid, and the epidermal cells at the four corners of the anther were larger in volume, containing thick cytoplasmic archesporial cells.

Sporogenous cell stage (**Figures 2A-B**): The cells of the meristematic tissue differentiated into sporogenous cells, characterized by thick nuclei and strong dividing ability. The cells at the four corners of the anther divided rapidly and expanded outward, making the anther tetragonal. The archesporial cells underwent one periclinal division and several anticlinal divisions, resulting in two layers: the outer primary parietal cells and the inner primary sporogenous cells.

Microspore mother cell stage (**Figures 2A-C**): The primary sporogenous cells formed secondary sporogenous cells after several mitotic divisions. The cells in the middle of the anther divided and differentiated multiple times, forming vascular tissue and thin-walled cells, constituting the central connective tissue. In this stage, four axially symmetrical microsporangia were clearly distinguishable. The secondary sporogenous cells continued to develop into microspore mother cells, characterized by thick nuclei and thin cell walls. Anther wall development was completed in this stage and consisted of four concentric layers: epidermis, endothecium layer, middle layer, and tapetum, from the outside to the inside.

Microspore mother cell meiosis stage (**Figures 2A-D**): The microspore mother cells underwent meiosis, forming microspore dyads and subsequently microspore tetrads.

Mononuclear microspore stage (**Figures 2A-E**): The tetrads were encapsulated by callose, a phase that lasted for a relatively short stage. This was followed by callose degradation and the release of mononuclear microspores.

Mature pollen (male gametophyte) stage (**Figures 2A-F**): The cytoplasm of the mononuclear microspores vacuolated, leading to a rapid increase in size and gradual transition to a spherical shape. This was followed by two mitotic divisions. The first mitotic division marked the onset of male gamete development. During the second mitotic division, the cytoplasm split unevenly, forming a cell plate between the two nuclei. This resulted in one larger vegetative nucleus and two smaller generative nuclei, forming a tricellular pollen grain.

The SEM observations of mature pollen grains (**Figure 2B**) revealed that the mature pollen grains were generally uniform in size (**Figures 2B-A**). Most pollen grains were full in shape, whereas a small portion appeared shriveled and broken, probably because the vacuum environment of the SEM causes the pollen wall to rupture. The polar (**Figures 2B-B**) views showed that the pollen apex was protruding, with three germination grooves extending to the two poles. The pollen grains were trilobate-ovoid in shape. The average length of the equatorial axis was 15.5 ± 0.8 μm, and the length of the polar axis was 30 ± 1.5 μm, resulting in a polar/equatorial axis length ratio of approximately 1.93. The outer wall of the pollen grains exhibited coral-like tomentose ornamentation, and the pore holes beneath this ornamentation were filled with pores (**Figures 2B-C**).

**Figure 3:**
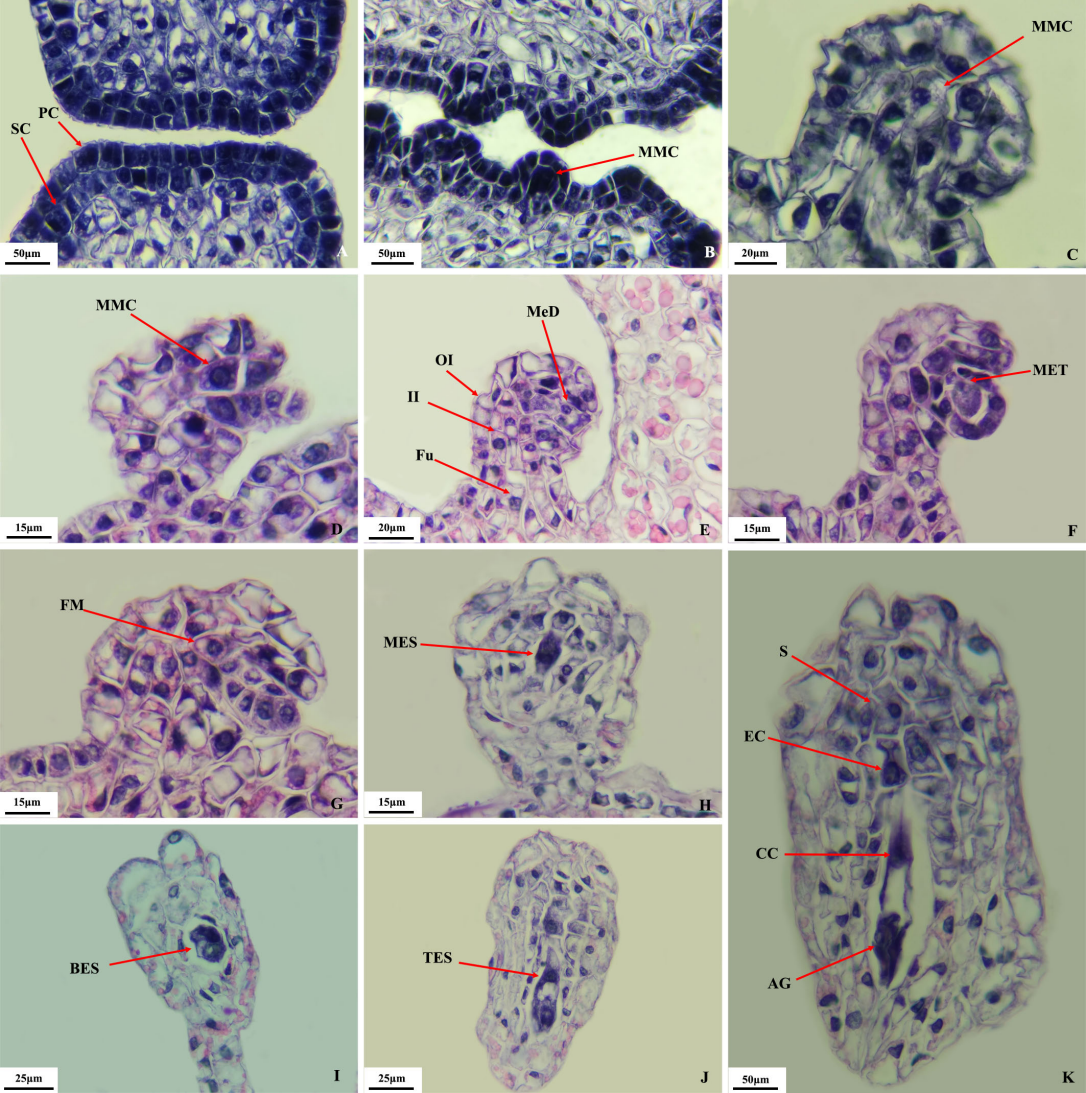
Megaspore development in *O. coerulescens*. **(A)** sporogenous cells stage; **(B–D)** megaspore mother cell stage; **(E, F)**, meiosis stage; **(G)** functional megaspore stage; **(H)** mononuclear embryo sac stagel; **(I)** binuclear embryo sac stage; **(J)** tetranuclear embryo sac stage; **(K)** Mature embryo sac stage. SC, sporogenous cell; PC, parietal cell; MMC, megaspore mother cell; Fu, funicle; II, inner integument; OI, outer integument; MeD, megaspore dyad; MeT, megaspore tetrad; FM, functional megaspore; MES, mononuclear embryo sac; BES, binuclear embryo sac; TES, tetranuclear embryo sac; AG, Antipodal group; CC, central cell; EC, egg cell; S, synergid.

Microscopic observation (**Figure 3**) showed that the pistil of *O. coerulescens* consisted of two carpels and one ovary with lateral membranous placentation. The ovule primordium, located in the inner chamber of the ovary, gave rise to the megaspore mother cells. These cells underwent one periclinal division and several anticlinal divisions to form two layers: the outer layer developed into the nucellus, and the inner layer contained the sporogenous cells (**Figure 3A**). The megaspores developed through the following stages.

Megaspore mother cell stage: As the nucellus developed, the sporogenous cells gradually increased in size and protruded outward, developing into megaspore mother cells (**Figures 3B–D**). Asymmetric growth of the funicle caused them to invert.
Megaspore mother cell meiosis stage: The megaspore mother cells underwent two consecutive meiotic divisions, forming dyads (**Figure 3E**) and tetrads (**Figure 3F**).
Mononuclear embryo sac stage: The functional megaspore (**Figure 3G**) at the chalazal end of the tetrad developed normally, whereas the three megaspores at the micropylar end degenerated, resulting in a mononuclear megaspore (**Figure 3H**). In this stage, the ovule was fully inverted.
Binuclear embryo sac stage: The functional megaspore underwent mitosis to form a binuclear embryo sac, with one nucleus near the chalazal end and another near the micropylar end (**Figure 3I**).
Tetranuclear embryo sac (TES) stage: Subsequent mitotic divisions of the nuclei at both ends resulted in a tetranuclear embryo sac (**Figure 3J**).

Mature embryo sac (female gametophyte) stage: In the early eight-nuclear stage, one nucleus from each end moved toward the center to form the central cell. The three nuclei at the chalazal end differentiated into antipodal cells, whereas the micropylar end differentiated into the egg apparatus, consisting of one egg cell and two synergids. This process was synchronized at both ends. The antipodal cells then divided several times to form a dense cluster. The mature embryo sac contained the antipodal cell cluster, the central cell, and the egg apparatus (**Figure 3K**). In summary, embryo sac formation belonged to the polygonum type.

The comparative morphological observation result of microspores and megaspores in *O. coerulescens* showed that (**Supplementary Figure S1**; **Supplementary Table S1**) the differentiation of stamens occurred earlier than that of pistils, with the formation of microsporogenous cells occurring approximately 15 days before that of megasporogenous cells. The development of microspores was also earlier than megaspores, with male gametophytes maturing during the F5 stage, whereas female gametophytes maturing during the F6 stage.

### 3.3 Physiological and biochemical changes during the development of microspores and megaspores

**Figure 4:**
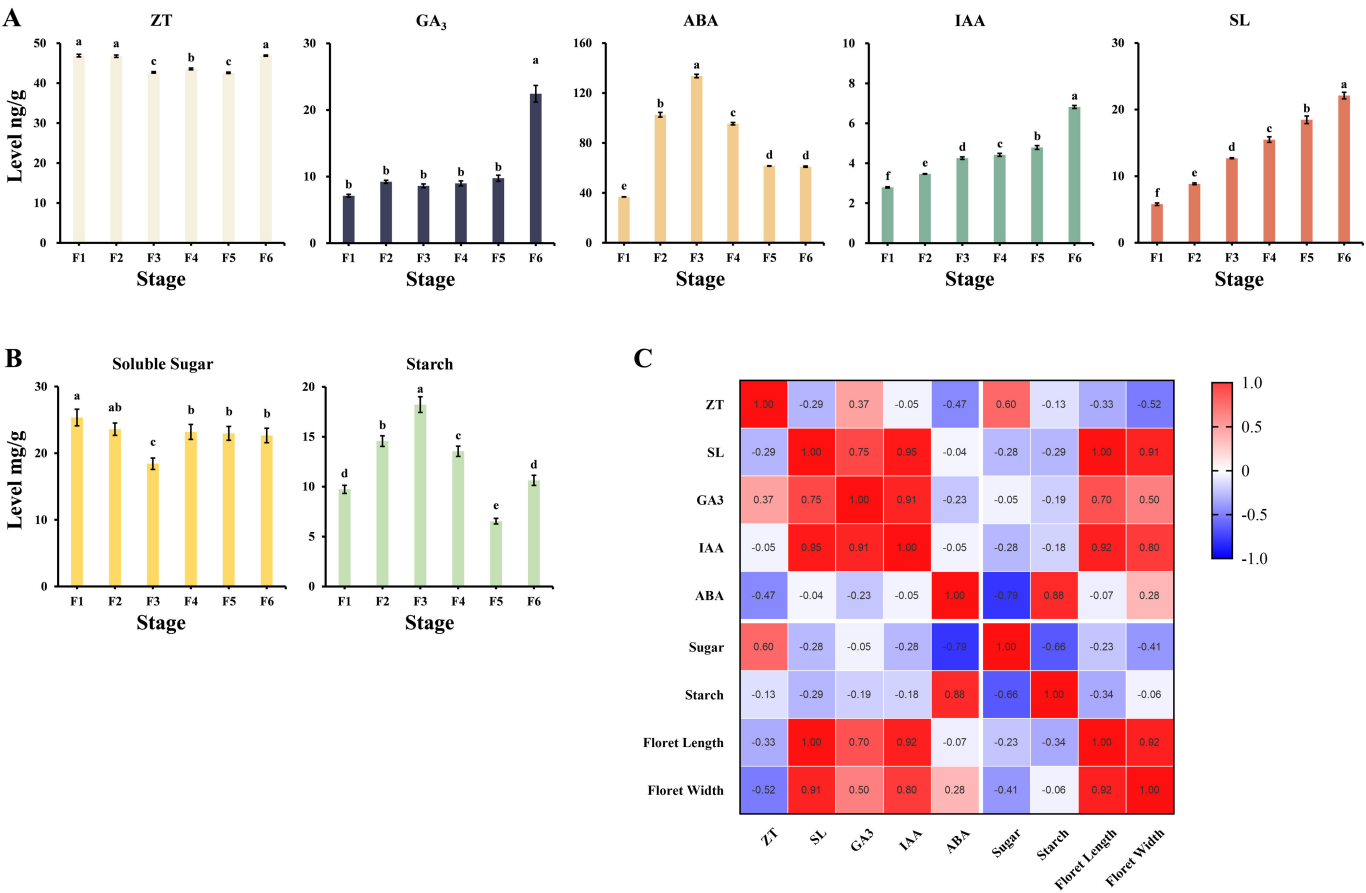
**(A)** Phytohormone levels at different developmental stages of microspores and megaspores in *O. coerulescens*, the x-axis represents different developmental stage and the y-axis represents phytohormone levels. **(B)** Sugar and starch levels at different developmental stages of microspores and megaspores in *O. coerulescens*, the x-axis represents different developmental stage and the y-axis represents sugar and starch levels. Data represents the mean ± SD, each group has three biological replicates. Letters above each box indicate statistical significance(*p<0.05*) as determined by ANOVA with Tukey HSD (for GA, ABA, ZT and sugar) and Kruskal-Wallis with Dunn’s test (for IAA, SL and starch). **(C)** Map of correlation between *O. coerulescens* flower morphology indicators and physiological indicators. The numbers in the box represent Spearman’s correlation coefficients. In the grid, the redder the color, the higher the positive correlation, while the bluer the color, the higher the negative correlation.

Level analyses were performed in six developmental stages separately to investigate the differences in phytohormones and sugars during the development of microspores and megaspores. The levels of zeaxanthin (ZT), SL, abscisic acid (ABA), IAA, and gibberellin (GA) showed different change patterns during the development of microspores and megaspores (**Figure 4A**). SL and IAA demonstrated progressive accumulation from F1 to F6 stage. ZT maintained homeostatic levels during F1 to F2 stage, followed by oscillatory fluctuations at later stages while generally maintaining a high overall level. ABA gradually increased to the highest point from F1 to F3 stage and then showed a significant downward trend. GA did not change significantly from F1 to F5 stage but surged at F6 stage. Carbohydrate metabolism displayed reciprocal accumulation patterns (**Figure 4B**), soluble sugar showed a decreasing trend from F1 to F3 stage and increased to a stable level after F4 stage. Starch accumulated during F1 to F3 stage, subsequently showing a significantly decreasing and then increasing trend. Spearman’s correlation analysis (**Figure 4C**) revealed that the SL, GA, and IAA levels showed a highly positive correlation with the floret size, whereas the ABA and ZT levels exhibited a moderately negative correlation. Sugar and starch levels are also moderately negatively correlated, suggesting their regulatory interplay in resource allocation.

### 3.4 Identification and functional enrichment of differentially expressed genes during the development of microspores and megaspores

Transcriptome sequencing was used to generate 18 transcriptome libraries from 6 groups representing different developmental stages of *O. coerulescens* flowers (microspores and megaspores), with 3 biological replicates per group. The percentage of Q20 bases in the high-quality clean data exceeded 98.15%, whereas Q30 bases exceeded 94.74%, and the GC content ranged from 44.81% to 45.37%. These results indicated that the sequencing data were reliable and suitable for further transcriptomic analysis. After assembling the clean reads into unigenes using Trinity software (http://trinityrnaseq.sourceforge.net), 108,170 unigenes from all samples were annotated using the (Non-Redundant Database) NR, (Kyoto Encyclopedia of Genes and Genomes) KEGG, (euKaryotic Orthologous Groups) KOG, and Swiss-Prot databases through Blastx. The numbers of annotated unigenes were then counted for each database. A total of 37,290 unigenes were genetically annotated, accounting for 34.5% of the total, indicating that much more genetic information of the parasitic *O. coerulescens* remains to be explored.

**Figure 5:**
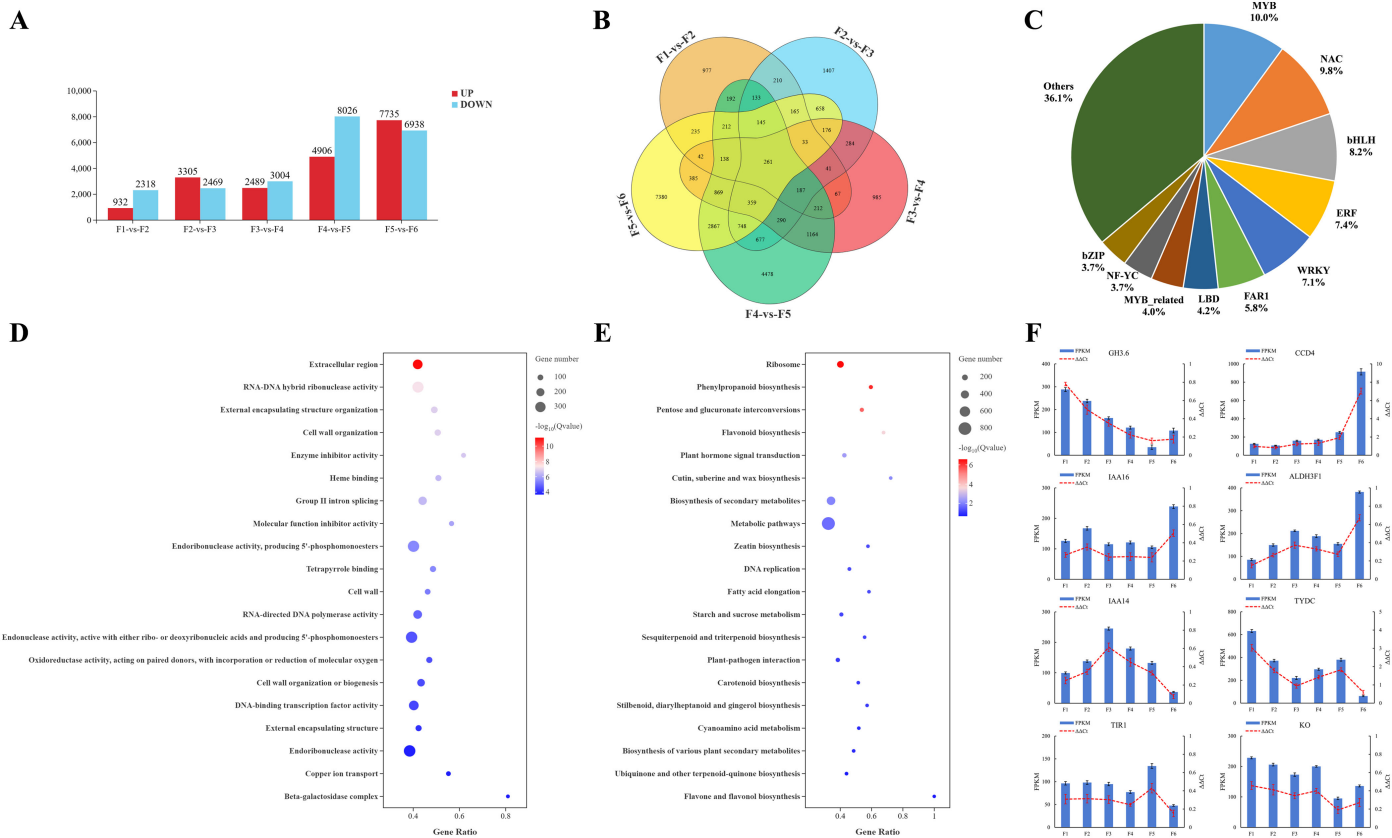
Identification and functional enrichment of DEGs at different developmental stages of microspores and megaspores in *O. coerulescens*. **(A)** Overview of upregulated and downregulated genes, red bars represent up-regulated genes and blue bars represent down-regulated genes; **(B)** Venn diagram of DEGs in different comparison groups; **(C)** First 10 TF families in the DEGs; **(D)** GO enrichment analysis for the DEGs, larger bubbles represent more DEGs enriched to GO terms, and darker red color represents more significant enrichment; **(E)** KEGG enrichment analysis for the DEGs, larger bubbles represent more DEGs enriched into the Kegg Pathway, and darker red color represents more significant enrichment; **(F)** qRT-PCR validation of 8 DEGs. The x-axis represents different developmental stages while the left y-axis represents the FPKM value of each gene and right y-axis represents the relative mRNA expression.

A comparative transcriptomic analysis was performed using the DEseq2 software (http://www.bioconductor.org/packages/release/bioc/html/DESeq2.html) to investigate the expression patterns of the differentially expressed genes (DEGs) throughout the flower development (**Figure 5A**). A total of 3250 DEGs were identified in the F1 stage compared with the F2 stage, with 932 upregulated genes and 2318 downregulated genes. Comparing the F2 stage with the F3 stage, 5774 DEGs were identified, of which 3305 were upregulated and 2469 were downregulated. Between the F3 and F4 stages, 5493 DEGs were found, of which 2489 were upregulated and 3004 were downregulated. In the F4 stage compared with the F5 stage, 12,932 DEGs were found, of which 4906 were upregulated and 8026 were downregulated. Finally, the F5 stage compared with the F6 stage showed 14,673 DEGs, of which 7735 were upregulated and 6938 were downregulated.

The Venn diagram analysis (**Figure 5B**) of DEGs at different stages of spore development showed that 977, 1407, 985, 4478, and 7380 genes were uniquely expressed in F1-VS-F2, F2-VS-F3, F3-VS-F4, F4-VS-F5, and F5-VS-F6, respectively. Additionally, 261 genes were differentially expressed across all stages.

Based on these analyses, 379 DEGs were related to 34 TF families (**Figure 5C**), among which the top 10 TF families were that of MYB (38, 10.03%), followed by NAC (37, 9.76%), bHLH (31, 8.18%), ERF (28, 7.39%), WRKY (27, 7.12%), FAR1 (22, 5.80%), LBD (16, 4.22%), MYB_related (15, 3.96%), NF-YC (14, 3.69%), and bZIP (14, 3.69%). We also conducted (Gene Ontology) GO and KEGG enrichment analyses, and the results showed that all the DEGs were divided into 49 level-2 GO functional classification terms (**Supplementary Figure S2**), including 27 biological processes, 19 molecular functions, and 3 cellular components. The first three terms corresponding to biological processes were cellular process, metabolic process, and biological regulation. Among the molecular function terms, the majority of genes were classified into binding, catalytic activity, and transporter activity. In addition, terms related to cellular components were mainly involved in cellular anatomical entity and protein-containing complex. The most significantly enriched GO terms (**Figure 5D**) included the extracellular region, RNA–DNA hybrid ribonuclease activity, external encapsulating structure organization, cell wall organization, and enzyme inhibitor activity. In KEGG analysis (**Supplementary Figure S3**), all DEGs were divided into 104 metabolic pathways, 21 genetic information processing pathways, 4 environmental information processing pathways, 2 organic system pathways, and 4 cellular process pathways. Among these, ribosome, phenylpropanoid biosynthesis, pentose and glucuronate interconversion, plant hormone signal transduction, zeatin biosynthesis, starch and sucrose metabolism, and tryptophan metabolism were significantly GO-enriched pathways (**Figure 5E**).

To further validate the accuracy of the sequencing results for differentially expressed genes at different developmental stages in *O. coerulescens*, four differentially expressed genes related to endogenous hormone synthesis (*CCD4*, *ALDH3F1*, *TYDC*, and *KO*) and four differentially expressed genes related to endogenous hormone signaling (*GH3.6*, *IAA16*, *IAA14*, and *TIR1*) were selected from the RNA-seq results and validated by qRT-PCR. The qRT-PCR results (**Figure 5F**) showed that the relative gene expression trends of the eight genes obtained by qRT-PCR were generally consistent with the RNA-seq results, further demonstrating the accuracy of the transcriptome sequencing results.

Through KEGG pathway annotation, relevant enzyme gene information for the main pathways, the expression patterns of DEGs in starch and sucrose metabolism, phytohormone synthesis, and phytohormone signaling in *O. coerulescens* were constructed.

**Figure 6:**
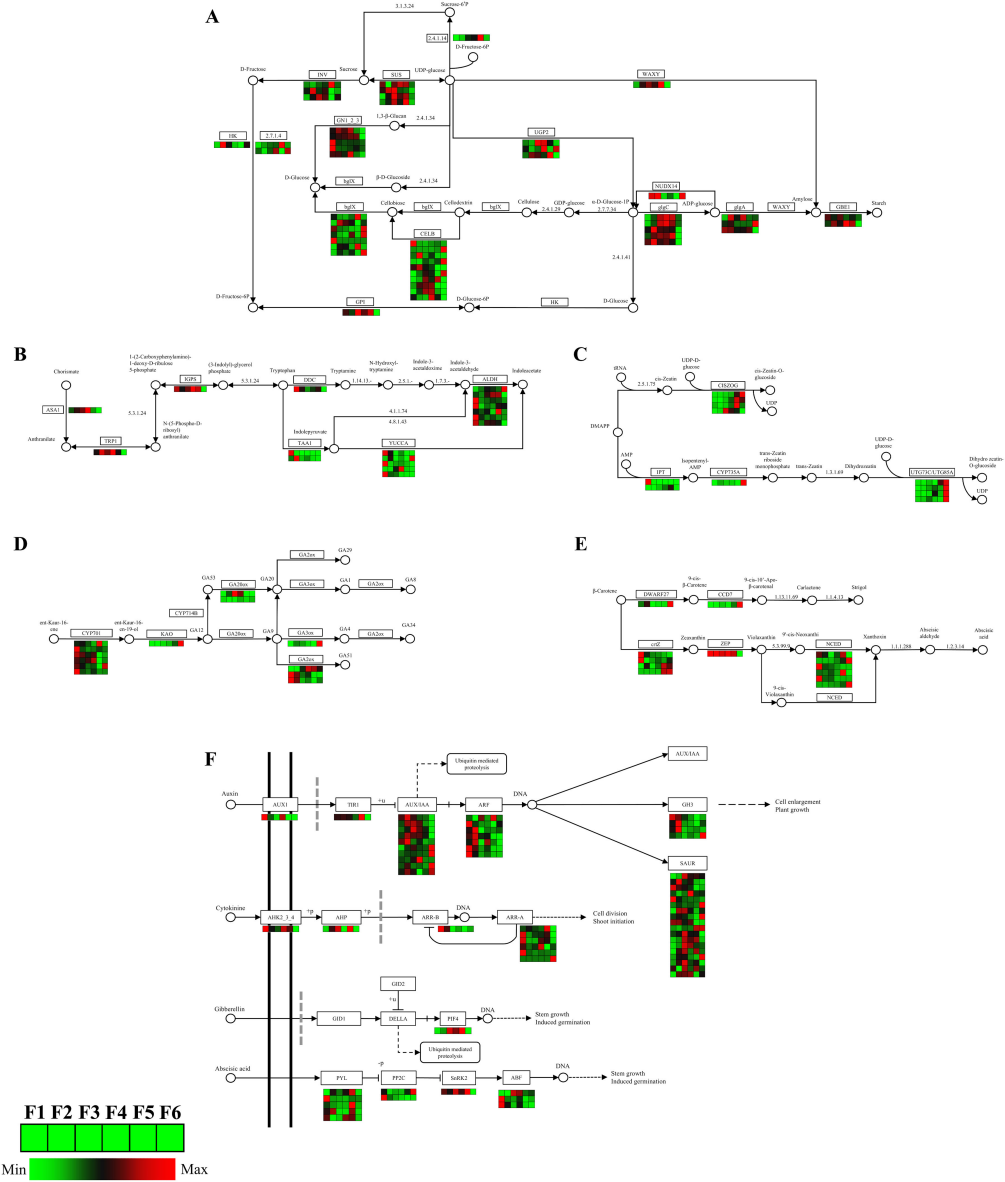
Analysis of DEGs related to energy metabolism, phytohormone synthesis, and signaling pathway. **(A)** Starch and sucrose metabolism pathway; **(B)** auxin synthesis pathway; **(C)** zeatin synthesis pathway; **(D)** gibberellins synthesis pathway; **(E)** abscisic acid synthesis pathway; and **(F)** phytohormone signaling pathway. In the grid, the redder the color, the higher the gene expression level, while the greener the color, the lower the gene expression level.

The starch and sucrose pathway (**Figure 6A**) had 49 DEGs; the coding genes of sucrose synthase (*SUS*) related to sucrose synthesis were mostly expressed at their highest levels during the F3 stage. The DEGs related to D-fructose synthesis showed that the genes encoding hexokinase (*HK*) had the highest expression level during the F2 stage. The encoding genes of nudix hydrolase homolog 14 (*NUDX14*) related to starch synthesis were highly expressed in the early and late stages of development, whereas the encoding genes of glucose-1-phosphate adenylyltransferase (*glgC*) were mostly highly expressed in the middle stages of development.

The IAA synthesis pathway (**Figure 6B**) had 18 DEGs. During the synthesis of tryptophan, a precursor substance of auxin, genes encoding anthranilate synthase alpha subunit 1 (*ASA1*), tryptophan biosynthesis 1 (*TRP1*), and Indole-3-glycerol phosphate synthase (*IGPS*) had high expression levels in the mid-stage. During auxin synthesis, the genes encoding L-tryptophan–pyruvate aminotransferase 1 (*TAA1*) and aromatic-L-amino-acid/L-tryptophan decarboxylase (*DDC*), as well as most genes encoding indole-3-pyruvate monooxygenase (*YUCCA*) and aldehyde dehydrogenase (*ALDH*), had the highest expression levels during the F1 stage.

The ZT synthesis pathway (**Figure 6C**) had 11 DEGs. The gene encoding adenylate dimethylallyl transferase (*IPT*) had the highest expression level at the F1 stage, and the genes encoding cis-zeatin O-glucosyltransferase (*CISZOG*), cytokinin trans-hydroxylase (*CYP735A*), and UDP-glucosyltransferase 73C (*UTG73C*) had the highest expression levels at the F5 or F6 stage.

The GA synthesis pathway (**Figure 6D**) had 16 DEGs. Most of the genes encoding ent-kaurene oxidase (*CYP701*) and gibberellin 2beta-dioxygenase (*GA2ox*) had the highest expression levels during the F1 stage, the gene encoding gibberellin 20-oxidase (*GA20ox*) had the highest expression level during the F3 stage, and those encoding gibberellin 3beta-dioxygenase (*GA3ox*) and ent-kaurenoic acid monooxygenase (*KAO*) had the highest expression levels during the F6 stage.

The SL and ABA synthesis pathways (**Figure 6E**) had 13 DEGs. The genes encoding beta-carotene isomerase (*DWARF27*) and 9-cis-beta-carotene 9’,10’-cleaving dioxygenase (*CCD7*) had the highest expression levels during the F6 stage, those encoding beta-carotene 3-hydroxylase (*crtZ*) had the highest expression during both the F1 and F6 stages, the gene encoding zeaxanthin epoxidase (*ZEP*) had high expression from F1 to F5 stage, and those encoding 9-cis-epoxycarotenoid dioxygenase (*NCED*) had high expression during the F1, F5, and F6 stages.

The phytohormone signaling pathway (**Figure 6F**) had 62 DEGs. The IAA signaling pathway had 41 DEGs. The genes encoding Transport Inhibitor Response 1 (*TIR1*), auxin influx carrier (*AUX1*), and auxin-responsive protein IAA (*AUX/IAA*) had the highest expression levels during the F5 stage, F1 stage, and the F2, F3, F4, and F6 stages, respectively. Most genes encoding auxin response factor (*ARF*) and Auxin-responsive GH3 family protein (GH3) had the highest expression levels during the F1 and F2 stages. The cytokinin (CTK) signaling pathway had nine DEGs. The genes encoding arabidopsis histidine kinase 2/3/4 (*AHK2_3_4*), two-component response regulator ARR-B family (*ARR-B*) and two-component response regulator ARR-A family (*ARR-A*) mostly expressed the highest levels during the F1 stage. The gene encoding histidine-containing phosphotransfer protein (*AHP*) had the highest expression level during the F5 stage.

The GA signaling pathway had only 1 DEG, which encoded the phytochrome-interacting factor 4 (*PIF4*) gene, and expressed the highest level during the F5 stage.

The ABA signaling pathway had 11 DEGs. The genes encoding abscisic acid receptor PYR/PYL family (*PYL*) mostly expressed the highest levels during the F5 stage, those encoding protein phosphatase 2C (*PP2C*) had the highest expression level during the F1 or F6 stage, and those encoding serine/threonine-protein kinase SRK2 (*SnRK2*) had the highest expression level during the F5 stage. Most genes encoding ABA responsive element binding factor (*ABF*) had the highest expression levels during the F1 stage.

### 3.5 Co-expression network analysis and identification of phytohormone synthesis and signaling genes

**Figure 7:**
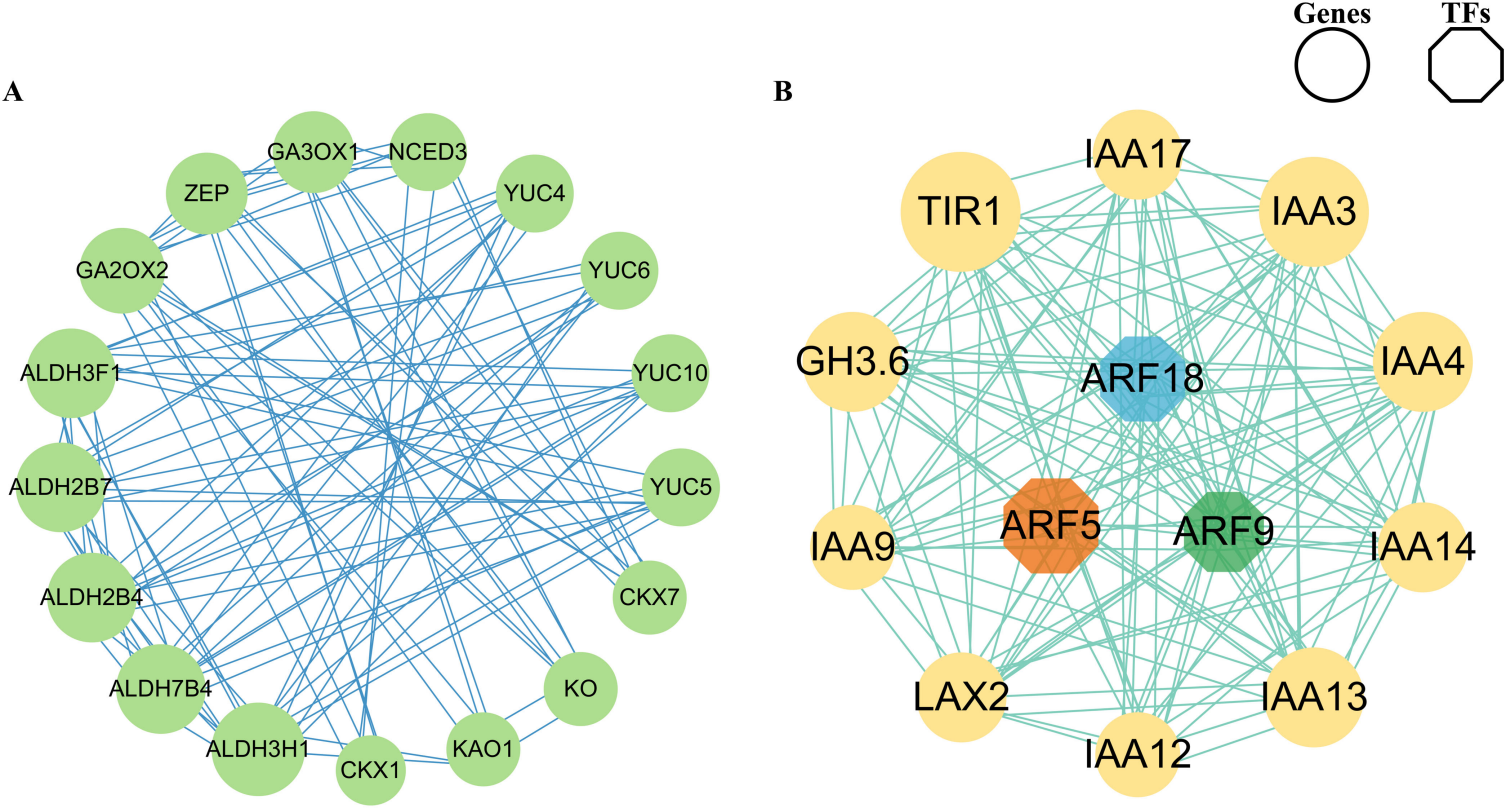
Co-expression network of identified TFs and genes. **(A)** Co-expression network of phytohormone synthesis DEGs. **(B)** Co-expression network of phytohormone signal DEGs. The size of the circle refers to the interaction strength with other nodes.

The co-expression network was constructed using the homologous proteins in *Arabidopsis* in the STRING (Search Tool for Recurring Instances of Neighboring Genes) database and visualizing the network using Cytoscape software to identify the regulatory relationships between phytohormone synthesis and phytohormone signal genes. The first network (**Figure 7A**) showed that five genes including Unigene0052998, Unigene0007531, Unigene0018251, Unigene0064915, and Unigene0112850 encoding *ALDH3H1*, *ALDH3F1*, *ALDH7B4*, *ALDH2B4*, and *ALDH2B7* separately showed strong interaction with other genes, implying that these may play a crucial role in regulating phytohormone synthesis during microspores and megaspores development in *O. coerulescens.* The second network (**Figure 7B**) showed that two genes including Unigene0026342 and Unigene0062820 encoding *TIR1* and auxin-responsive protein (*IAA3*) showed strong interaction with other genes, implying that these may play a crucial role in phytohormone signaling. In addition, we found that Unigene0057628 encoding auxin response factor 5 (*ARF5*) showed the strongest interaction with other genes and could therefore be considered the hub regulators.

## 4 Discussion

The parasitic behavior of the *Orobanche* genus represents a global agricultural crisis, affecting a wide range of crops and causing significant production losses. However, *Orobanche* species play an irreplaceable role in natural ecosystems by regulating community biodiversity, increasing nutrient cycling, and promoting ecological succession. Moreover, these plants hold high medicinal value due to their abundance of various natural secondary metabolites. Understanding the development processes of microspores and megaspores in *O. coerulescens* is crucial for elucidating their sexual reproduction mechanisms, which can aid in the sustainable utilization of *O. coerulescens*. This study aimed to comprehensively analyze the regulatory mechanisms underlying microspore and megaspore development in *O. coerulescens* by examining morphological changes, physiological and biochemical traits, and transcriptomic data, thereby contributing to research on reproductive genetics in this species.

### 4.1 Microspore and megaspore development in *O. coerulescens*

Sexual reproduction in angiosperms is vital in ensuring genetic diversity, species adaptability, and ecological stability (Mangla et al., 2023), and the development of microspores and megaspores is essential for sexual reproduction. This intricate process ensures genetic diversity and successful reproduction in flowering plants.

In this study, microscopic and SEM observations (**Figure 2**) of *O. coerulescens* exhibited four stamens, of which two were strong, and each anther had four microsporangia. The ovary had two carpels. Pollen matured about 15 days earlier than ovules. In the maturation stage, a large amount of pollen and ovules were present. Starting from the end of February, the development of the microspores could be divided into six stages: Archesporial cell stage, sporogenous cell stage, microspore mother cell stage, microspore mother cell meiosis stage, mononuclear microspore stage, and mature pollen (male gametophyte) stage. The anther wall was fully developed in the microspore mother cell stage and consisted of the epidermis, endothecium, middle layer, and tapetum. At the end of mitosis, the tapetum was completely degraded and the middle layer was compressed, leaving only a linear residue. Mature *O. coerulescens* pollen was tricellular, with three long bipolar germination grooves, an overall trilobate-ovoid shape, and a tomentose surface ornamented with burrows. The developmental process of *O. coerulescens* microspores was similar to other tricellular pollen plants, such as *Oryza sativa* (rice) and *Arabidopsis* (Zhang et al., 2011; D’Ippólito et al., 2017), and the surface structure of pollen was consistent with previous studies (Piwowarczyk et al., 2015).

The developmental process of microspores and megaspores in *O. coerulescens* lasted approximately 2 months. The development of megaspores (**Figure 3**) in *O. coerulescens* spanned seven stages: sporogenous cell stage, megaspore mother cell stage, megaspore mother cell meiosis stage, mononuclear embryo sac stage, binuclear embryo sac stage, tetranuclear embryo sac stage, and mature embryo sac (female gametophyte) stage. Ovules begin to invert in the megaspore mother cell stage and complete in the mononuclear embryo sac stage. The formation of the mature embryo sac belonged to the typical monosporangiate polygonum type. *O. coerulescens* broke the ground at the end of February each year, and the process of flower development lasted about 2 months. The comparative morphological observation result of microspores and megaspores in *O. coerulescens* showed that (**Supplementary Figure S1**; **Supplementary Table S1**) the microspores developed and matured about 15 days earlier than the macrospores. The timing difference in the maturation of male and female gametophytes requires further investigation to determine its impact on self-pollination.

Studying the morphological development of microspores and megaspores is essential for understanding plant reproductive biology. The development of these reproductive units is crucial for successful fertilization and subsequent generation propagation. Morphological variations often reflect underlying genetic and environmental influences, which are key in population maintenance and plant resilience to stressors. Moreover, understanding these developmental patterns aids in elucidating evolutionary adaptations in plant reproduction, highlighting how different plant species have optimized their reproductive strategies to thrive in diverse ecosystems.

### 4.2 Effect of carbohydrate metabolism on microspore and megaspore development in *O. coerulescens*

Sugar and starch play fundamental roles in plant sexual reproduction, serving as primary energy sources and structural components during the development of reproductive organs. In the male reproductive process, starch accumulates in developing pollen grains, providing the energy required for pollen maturation and germination. Similarly, in female reproductive structures, sugars and starches accumulate in ovules, supporting the embryo sac development and providing energy for fertilization and early seed development (Lakehal et al., 2019; Guo et al., 2020). Studies on *C. australis* have shown that starch degradation in the basal stems releases sucrose, which is transported to shoot tips to fuel elongation and host-seeking behavior, a critical step in parasitic plant reproduction (Zhang et al., 2022). In the starch and sucrose metabolic pathways, the coding genes for *SUS* are mostly expressed at their highest levels in the F3 stage. *SUS* is a pivotal enzyme in plant carbohydrate metabolism, facilitating the reversible conversion of sucrose and Uridine 5’-diphosphate (UDP) into UDP-glucose and fructose. In starch synthesis, SUS-derived UDP-glucose is converted into ADP-glucose, the direct substrate for starch synthase enzymes, thereby linking sucrose metabolism to starch accumulation (Stein and Granot, 2019). The encoding genes of *NUDX14* related to starch synthesis are highly expressed in the early and late stages of development, whereas the encoding genes of *glgC* are mostly highly expressed in the middle stages of development. *NUDX14* plays a significant role in the starch and sucrose metabolism pathway by hydrolyzing ADP-glucose, a key precursor in starch biosynthesis. The overexpression of *NUDX14* has been shown to reduce starch accumulation in rice, and the mutations in *NUDX14* lead to alterations in grain chalkiness, a trait associated with starch composition and structure (Liu et al., 2022). *glgC* is a pivotal enzyme that catalyzes the first committed step in starch biosynthesis; research has demonstrated that variations in *glgC* expression significantly influence starch and sucrose content in plants (Wu et al., 2024). Our findings demonstrate that sugar and starch play critical roles in the reproductive development of *O. coerulescens*. Dynamic fluctuations in their concentrations were observed across sequential stages of microspore and megaspore development, with the most pronounced shifts occurring during the meiosis phase. These metabolic changes may be associated with tapetal degeneration and tetrad callose degradation. In summary, the normal metabolism of sugar and starch is necessary for maintaining sexual reproduction in plants. Further research is needed to elucidate the underlying mechanisms of their effects in *O. coerulescens*.

### 4.3 Role of phytohormones during the development of microspores and megaspores in *O. coerulescens*

Phytohormones are signaling molecules that are vital in regulating plant growth, development, and responses to environmental stimuli, especially for spore formation and maturation (Suzuki et al., 2009; Gerashchenkov and Rozhnova, 2013; Zur et al., 2015). However, the regulatory mechanism of microspore and megaspore development in *O. coerulescens* remains unclear. In this study, IAA and SL levels significantly increase with the development process (**Figure 4**), suggesting their significance in microspore and megaspore development in *O. coerulescens*. *TAA1* and *YUCCA* are key enzymes in the simple two-step pathways of converting tryptophan to IAA in plants, which are crucial for the biosynthesis and regulation of auxin (Phillips et al., 2011; Zhao, 2012). In this study, two genes encoding *TAA1* and five genes encoding *YUCCA* were identified. Most of them were expressed at their highest levels in the F1 stage, thereby promoting the beginning of auxin accumulation. Meanwhile, *AUX1* is an auxin influx carrier involved in apical hook development in seedlings (Vandenbussche et al., 2010); one coding gene was identified, which was also expressed at the highest level during the F1 stage, further promoting the accumulation of IAA.

SL is a newly discovered carotenoid-derived phytohormone, which functions both as exogenous signals in the paraphyton host rhizosphere and as endogenous phytohormones (Walker et al., 2019). Although relatively few studies have focused on the role of SL in plant sexual reproduction, these studies showed that SL may play a key role in regulating floral organ development, seed development, and the symbiotic relationship between plants and fungi. Exogenously applied GR24, a synthetic SL analog, has been found to affect somatic embryo formation and morphogenesis in the SL receptor–deficient mutant tomato by interfering with IAA and CTK, suggesting that SL is involved in plant embryogenesis (Wu et al., 2017). In this study, the accumulation pattern of SL was similar to IAA, perhaps jointly regulating the development of microspores and megaspores in *O. coerulescens* with IAA. We identified a coding gene for *DWARF2*7, which was a non-mobile precursor upstream of More Axillary Branches 1 (*MAX1*), and a gene encoding the carotenoid cleavage dioxygenase *CCD7* in the SL synthesis pathway, with the highest expression levels in the F6 stage. The regulatory mechanism of microspore and megaspore development in *O. coerulescens* needs further investigation. ABA is a derivative of another branch of the carotenoid synthesis pathway, mainly known for its role in stress response. In contrast, it can also affect plant sexual reproduction through a complex gene regulatory network (Yang et al., 2022; Aerts et al., 2024). In this study, the ABA levels were the highest during mitosis. *PP2C* was a key negative regulatory factor in the ABA signaling pathway (Park et al., 2009); two identified coding genes were expressed at low levels during mitosis, indicating that ABA had a certain positive regulatory effect on the mitosis of *O. coerulescens.*

ZT participates in a series of sexual reproductive processes such as meiosis, intercellular communication between tapetum and microspores, and differentiation of megaspore mother cells (Hirano et al., 2008; Cai et al., 2023). In this study, the ZT level significantly decreased during the meiosis and mitosis stages. The encoding genes of two key enzymes, *IPT* and *CYP735A*, in its synthesis pathway had the highest expression levels in the early or late stages of microspores and megaspores development in *O. coerulescens*, consistent with the accumulation pattern of ZT.

GA is essential for the formation of the male meiotic cell wall and the maintenance of ploidy uniformity in the male gametophyte, controlling the number of ovules generated (Barro-Trastoy et al., 2022; Zhao et al., 2023). In this study, the GA level remained at a low level from the F1 to the F5 stage but sharply increased in the F6 stage. This change may be related to the high expression of genes encoding *KAO* and *GA3ox* in the F6 stage, the two key enzymes in the GA synthesis pathway. During the development process of microspores and megaspores in *O. coerulescens*, the levels of SL, IAA, and GA exhibited a significant positive correlation with progressive elevation. This coordinated hormonal pattern suggests their collective modulation of discrete physiological processes during both microsporogenesis and megasporogenesis, which synergistically contribute to the reproductive success of *O. coerulescens*. Nevertheless, the interplay underlying these phytohormonal dynamics requires further elucidation through comprehensive molecular investigations.

### 4.4 Co-expression analysis identified key DEGs of phytohormone synthesis and signaling

We established co-expression networks separately to find the most crucial genes in the network and identify the regulatory relationships between phytohormone synthesis and phytohormone signaling genes. In the phytohormone synthesis–related co-expression network (**Figure 7A**), we found that the coding genes of *ALDH3H1*, *ALDH3F1*, *ALDH7B4*, *ALDH2B4*, and *ALDH2B7* had the strongest interaction with other genes. *ALDHs* in plants play a crucial role in the biosynthesis of auxins, they oxidize aldehyde intermediates into carboxylic acids during the auxin biosynthesis process. Specifically, ALDH family enzymes are involved in converting indole-3-acetaldehyde into indole-3-acetic acid, the active form of auxin; it is a crucial step in the tryptophan-dependent auxin biosynthesis pathway, this reaction is essential for root elongation, shoot formation, fruit development, and response to environmental stimuli (Stiti et al., 2021; Raza et al., 2022). *OsALDH2b* (mitochondrial aldehyde dehydrogenase) was highly expressed in anthers from meiosis to the early microspore stage, negatively regulates tapetum degeneration in rice, the mutation of *OsALDH2b* leading to premature tapetum degeneration and abnormal microspore development (Xie et al., 2020).These provided further insight into the role of *ALDHs* in anthers development.

In phytohormone signaling–related co-expression network (**Figure 7B**), the coding genes of *ARF5* TFs of the ARF TF family had the strongest interaction with other genes, which may be key TFs regulating phytohormone signaling during the development of microspores and megaspores in *O. coerulescens*. The coding genes of *TIR1* and *IAA3* also had the strongest interaction with other genes. *ARF5* mediated the transmission of auxin signaling in stem cells and was involved in the transcriptional regulation of the entire Aux/IAA family in *A. thaliana* (Krogan et al., 2014; Luo et al., 2018). Silencing the *AtARF2-AtARF4* and *AtARF5* genes in *A. thaliana* can lead to identity defects in cells at the micropylar pole, as well as morphologically aberrant and unviable, loss of inclusions and nuclei, indicating that *ARF5* is essential for female and male gametophyte development (Liu et al., 2017). In the auxin signaling pathway, *TIR1* served as a central receptor facilitating the degradation of AUX/IAA proteins, such as *IAA3*, thereby enabling auxin-responsive gene expression. *IAA3* is a transcriptional repressor in the AUX/IAA family. It binds to ARFs to inhibit the activation of auxin-responsive genes. When auxin levels are high, *TIR1*-mediated degradation of *IAA3* removes this repression, allowing ARFs to initiate the transcription of genes involved in various developmental processes and differentiation (Lakehal et al., 2019; Shi et al., 2022; Jing et al., 2023). The subcellular localization of *GhTIR1* protein shows high expression levels in the flowers and early ovules of *Gossypium hirsutum*, indicating its importance for flower and ovule development (Wu et al., 2021). *TIR1* and *ARF5* primarily regulate plant embryonic development, floral organ formation, seed germination, and root development through the auxin signaling pathway, serving as core regulatory factors in plant reproductive strategies. *ALDHs* serve as critical guardians against oxidative cytotoxicity, mechanistically linked to the maintenance of gametophyte viability and embryogenesis, and enhancing plant adaptability to environmental stress. These genes work synergistically to ensure the successful completion of the reproductive process across different developmental stages and environmental conditions. Using exogenous hormones or genetic modification to regulate the expression of these key genes to control the development of microspores and megaspores in *O. coerulescens*, is an approach with dual applications in sustainable population management and targeted biocontrol strategies. At present, there is limited research on *ALDHs* in parasitic plants, and the functions and regulatory mechanisms in the reproductive development of parasitic plants need further exploration.

In summary, we investigated the development of microspores and megaspores in *O. coerulescens* by morphology, physiology and transcriptome, and identified the key genes for phytohormone synthesis as *ALDHs*, the key genes for phytohormone signaling as *TIR1* and *IAA3*, and the key TF as *ARF5*. This study enriches the basic information and dataset of the reproductive development in *O. coerulescens*, providing theoretical references for regulating its reproduction, implementing biological control measures, maintaining its population, and optimizing resource utilization.

## Data Availability

The datasets presented in this study can be found in online repositories. The names of the repository/repositories and accession number(s) can be found below: https://www.ncbi.nlm.nih.gov/bioproject/PRJNA1193147.
