# Supplementary material for: Morphophysiological and transcriptomic analyses during the development of microspores and megaspores in Orobanche coerulescens
Source: Front Plant Sci. 2025 Mar 26;16:1540594. doi: 10.3389/fpls.2025.1540594 (PMC11978843; doi:10.3389/fpls.2025.1540594)
Supplement: Supplementary file 1 [file DataSheet1.docx]

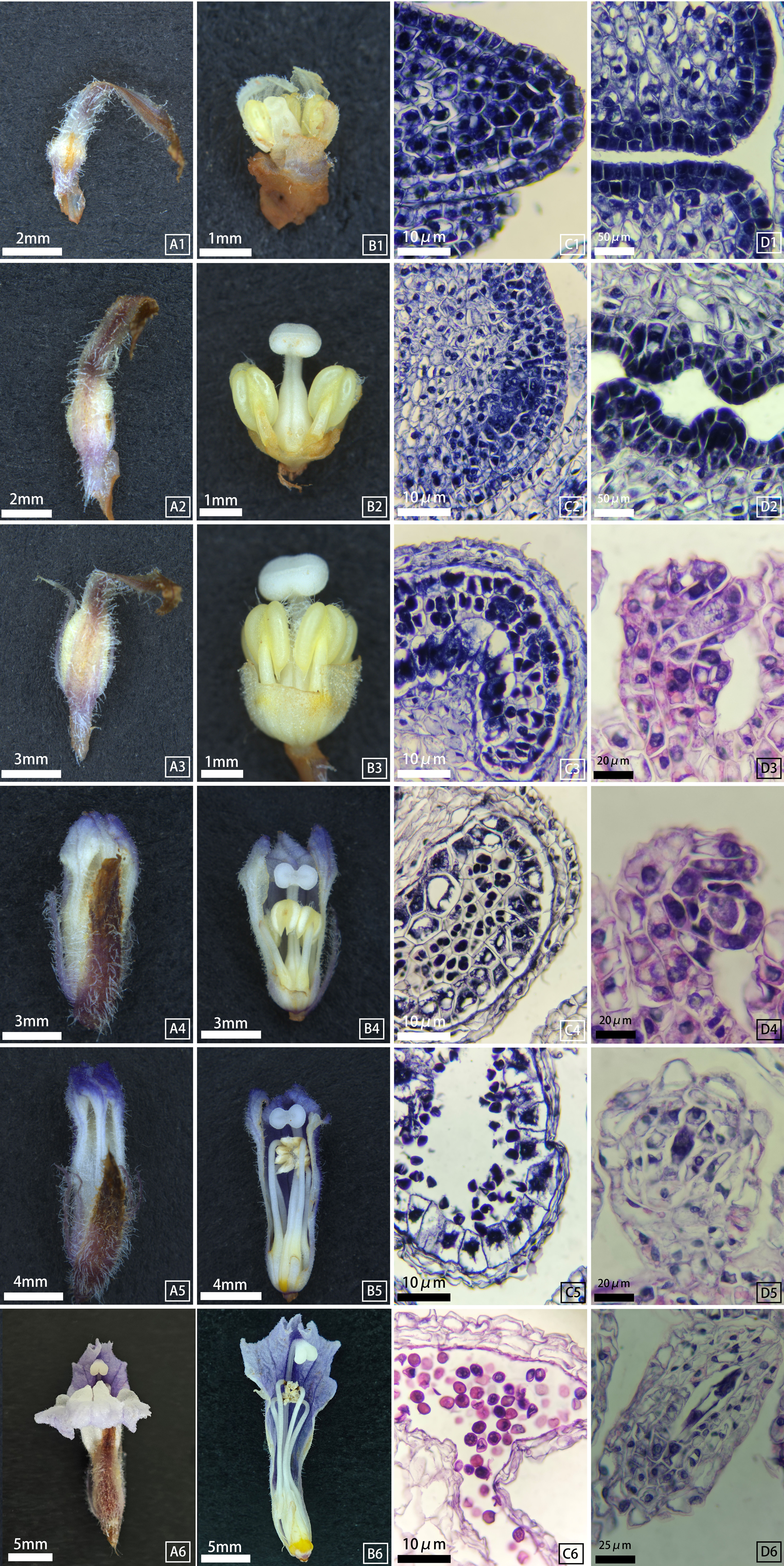


**Supplementary Figure 1.** The comparative morphological observation result of microspores and megaspores in *O. coerulescens*. A, External morphology of floret; B, External morphology of stamens and pistils; C, Microstructure of microspores; D, Microstructure of megaspores.


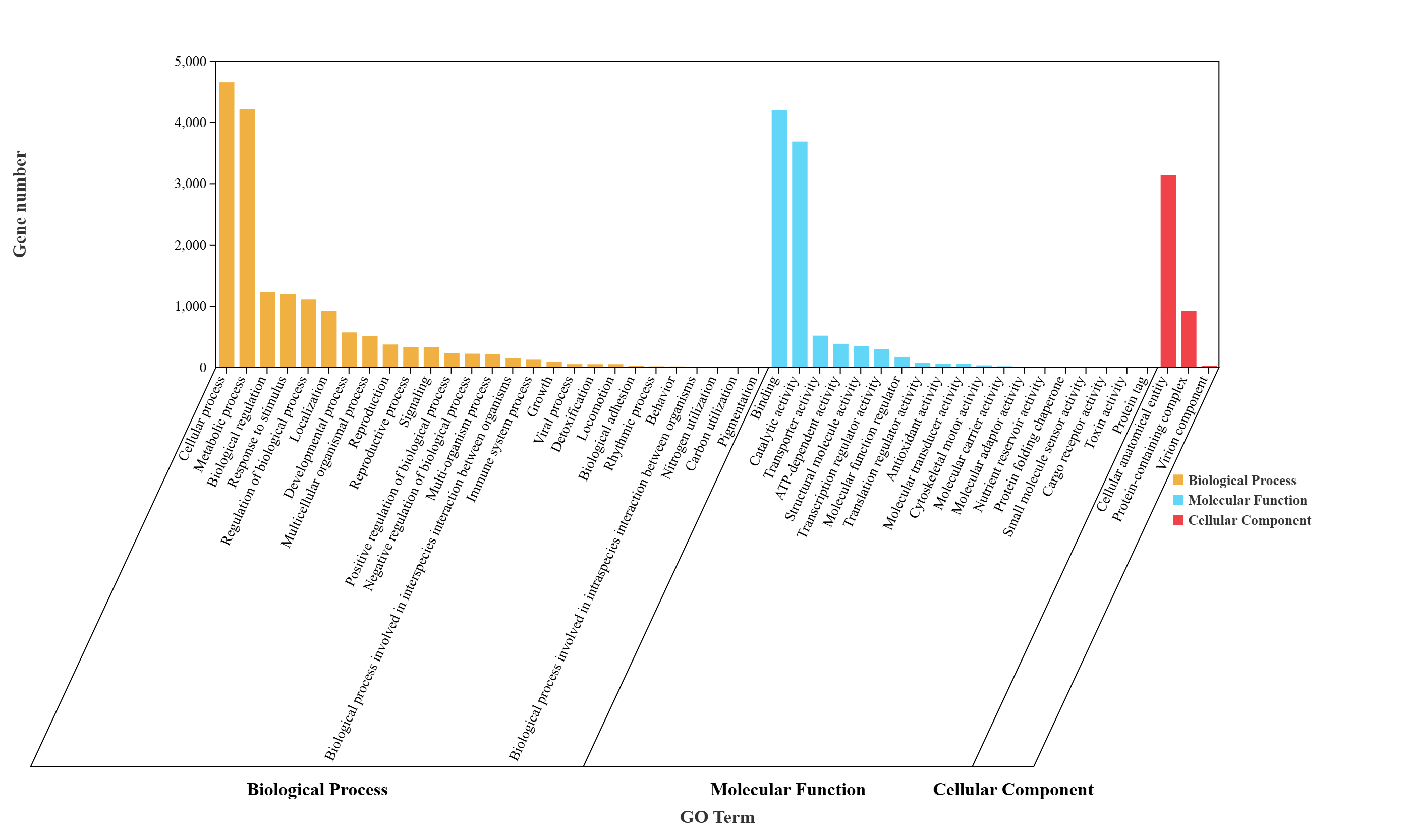


**Supplementary Figure 2.** Level-2 GO functional classification terms.


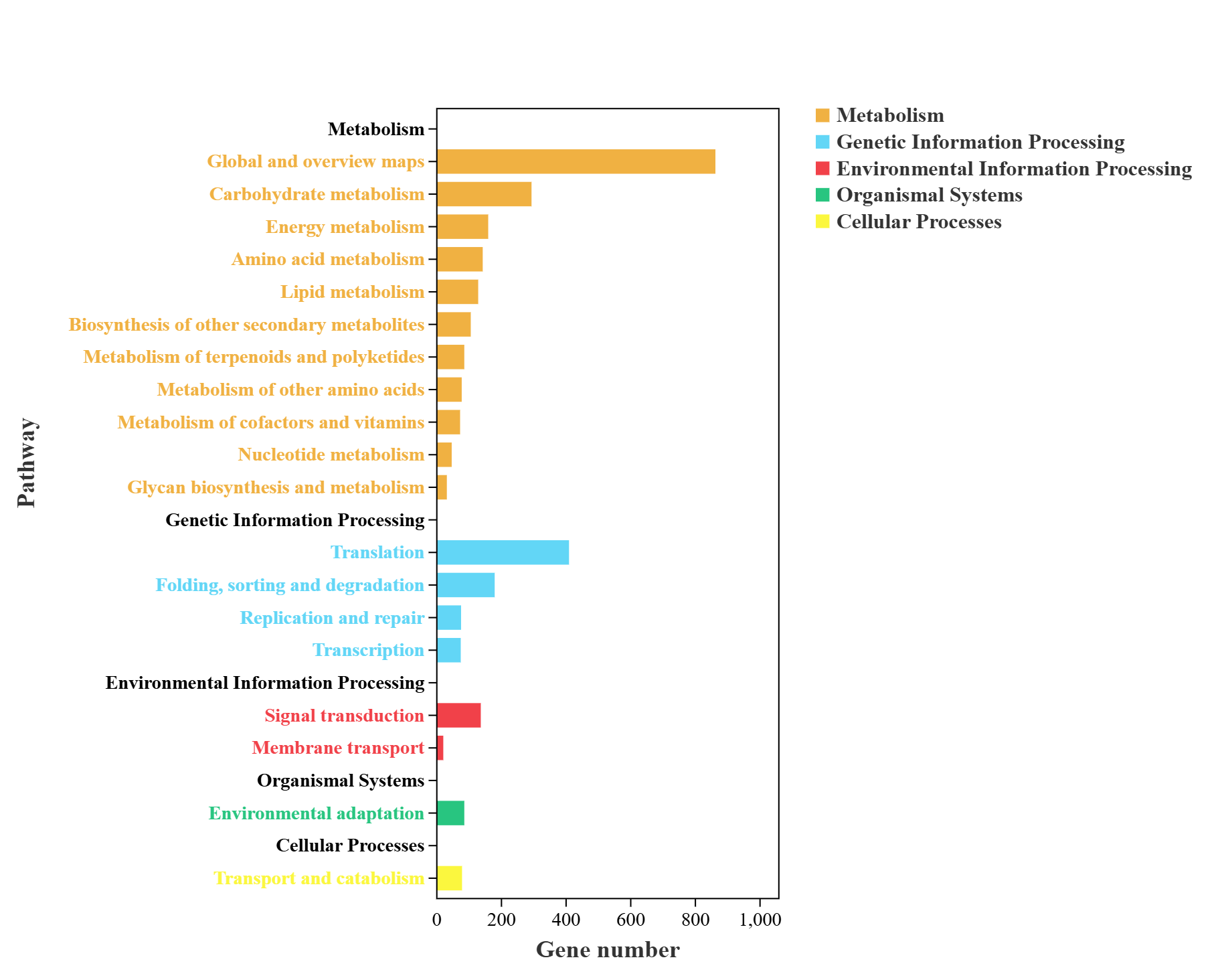


**Supplementary Figure 3.** Level-2 KEGG functional classification terms.
